# Inhibition of HIV-1 Replication and Dimerization Interference by Dual Inhibitory RNAs 

**DOI:** 10.3390/molecules15074757

**Published:** 2010-07-07

**Authors:** Francisco J. Sánchez-Luque, José A. Reyes-Darias, Elena Puerta-Fernández, Alfredo Berzal-Herranz

**Affiliations:** 1 Instituto de Parasitología y Biomedicina “López-Neyra”, CSIC, P.T. Ciencias de la Salud, Av. del Conocimiento s/n, Armilla, 18100 Granada, Spain; E-Mails: kiko@ipb.csic.es (F.J.S.-L.); joanreda@hotmail.com (J.A.R.-D.); 2 Instituto de Bioquímica Vegetal y Fotosíntesis, Universidad de Sevilla-CSIC. Américo Vespucio s/n, Isla de la Cartuja, 41092 Sevilla, Spain; E-Mail: elena.puerta@ibvf.csic.es (E.P.-F.)

**Keywords:** Ribozymes, antisense RNAs, HIV-1 RNA dimerization, anti-HIV RNAs

## Abstract

The 5’-untranslated region (5’UTR) of the HIV-1 RNA is an attractive target for engineered ribozymes due to its high sequence and structural conservation. This region encodes several conserved structural RNA domains essential in key processes of the viral replication and infection cycles. This paper reports the inhibitory effects of catalytic antisense RNAs composed of two inhibitory RNA domains: an engineered ribozyme targeting the 5’ UTR and a decoy or antisense domain of the dimerization initiation site (DIS). These chimeric molecules are able to cleave the HIV-1 5’UTR efficiently and prevent viral genome dimerization *in vitro*. Furthermore, catalytic antisense RNAs inhibited viral production up to 90% measured as p24 antigen levels in *ex vivo* assays. The use of chimeric RNA molecules targeting different domains represents an attractive antiviral strategy to be explored for the prevention of side effects from current drugs and of the rapid emergence of escape variants of HIV-1.

## 1. Introduction

The human immunodeficiency virus (HIV-1) is the etiological agent of AIDS [1]. It primarily targets CD4^+^ cells like T cells and macrophages, leading to the suppression of their immune function. The *quasi*-species structure of the viral population and its high mutation rate enable the virus to rapidly evade the immune response and promote the appearance of variants resistant to therapeutic drugs. Current therapies are based on a combination of different viral protein inhibitors that achieve a high reduction of the viral load, allowing a partial restoration of the immune function. Patients must maintain an aggressive lifelong treatment which produces serious side effects, so there is a real need to pursue the development of new therapies. Besides carrying the genetic information the HIV genomic RNA contains several conserved structural domains which play essential roles in viral replication and infection cycles. These functional RNA domains exhibit the highest sequence and structural conservations of the viral genome, making them very attractive therapeutic targets. The direct targeting of the viral RNA genome to interfere with the function of genomic RNA domains is a strategy to be explored, with nucleic acids being strong candidates as specific inhibitors for the development of this potential therapeutic technology. 

The HIV is a lentivirus which contains two long directly repeated regions at both ends of the genome. The 5’-untranslated region (5’UTR) of primary RNA transcripts comprises several functional RNA domains involved in key processes of the viral cycle like the trans-activation of transcription, polyadenylation, reverse transcription, dimerization, splicing and packaging [2]. The HIV infective particles contain two non-covalently linked genomic RNA copies that are bound through their 5’ ends. The dimerization process is initiated at the dimerization initiation site (DIS) stem-loop domain. The DIS contains a palindrome sequence motif within the apical loop which is involved in the initial homodimerization interaction by a kissing-loop mechanism which is thought to progress to a more extended interaction [3]. It has been proposed that the availability of the DIS structure is controlled by a riboswitch which alternates between two main 5’UTR conformers termed branched multiple hairpins (BMH) and long distance interaction (LDI) forms [4]. The existence of one or other of the conformational structures may determine the switch between full-length viral RNA packaging and translation processes [5]. *In silico* studies based on biochemical data support the notion that the LDI is the most abundant conformer [6], while *ex vivo* structure probing assays indicate the prevalence of the BMH conformer, *i.e.* the dimerizing-competent form [7]. Both conformers have been visualized in native gel electrophoresis using mutants that disrupt the equilibrium between both conformers [4]. 

**Figure 1:**
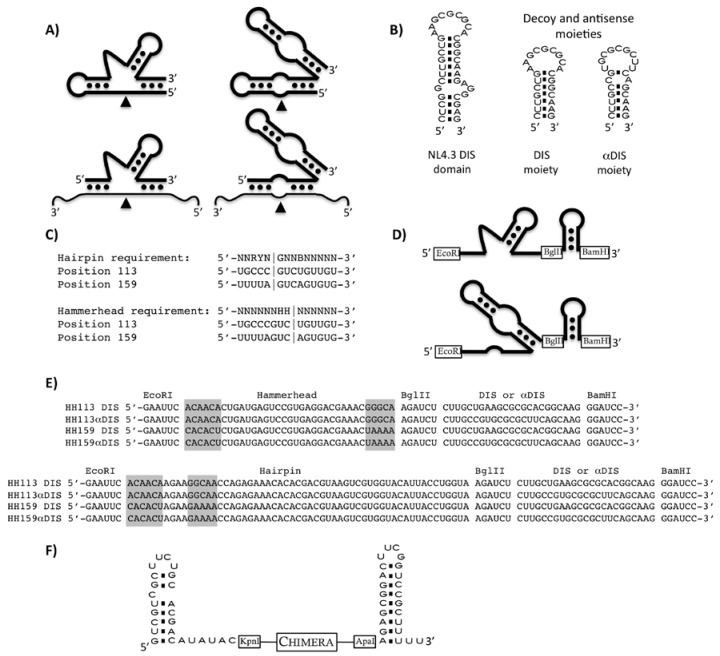
**(a)** Schematic representation of the catalytic RNAs. Self-cleaving hammerhead (top left) and hairpin (top right) ribozymes are represented. Engineered trans-cleaving ribozymes are represented at the bottom. The thinner line represents substrate RNA and the arrows indicate the cleavage points. **(b) **The wild type complete DIS domain of NL4.3 HIV-1 strain is shown on the left while the two inhibitory moieties used in this study, DIS sense and antisense (αDIS), are shown on the right. **(c)** Ribozyme sequence requirements and target sites 113 and 159 are indicated. Vertical lines represent the cleavage site. Nucleotide code: N stands for A, U, G or C; B stands for C, G or U; H stands for A, C or U; Y stands for C or U; and R stands for A or G. **(d)** Schematic representation of the catalytic chimera RNA series used in the *in vitro* experiments. The ribozyme domain coding sequences were flanked by *Eco*RI and *Bgl*II sites, while the decoy or antisense DIS domain were flanked by *Bgl*II and *Bam*HI sites. **(e)** Sequences of all combinations of chimeric RNAs used in this study. Grey boxes indicate the substrate recognition arms of the ribozyme domains. **(f)** Schematic representation of the chimeras used in the *ex vivo* inhibition assays, inhibitor RNAs are flanked by U6 snRNA end hairpin loops.

The minimal catalytic domains of the natural hammerhead and hairpin ribozymes have been engineered to generate trans-cleaving catalytic RNAs (Figure 1A) [8,9,10], and their substrate sequence requirements have been defined [11,12,13,14]. The therapeutic potential of the hammerhead and the hairpin catalytic motives has been widely proven [15]. We have previously shown efficient HIV-1 RNA inhibition by using inhibitor RNAs based on either hairpin or hammerhead ribozymes [16,17].

We report here the characterization of a series of chimeric molecules composed of a trans-cleaving ribozyme, either a hairpin or a hammerhead, targeting two different sites within the HIV-5’UTR and an anti-DIS specific molecule, either a DIS sense molecule that may act as a decoy element and an antisense DIS molecule (Figure 1B). *In vitro *analysis demonstrated that both sense and antisense-DIS domains interfere with viral dimerization. The antiviral activity was evaluated by transfecting cells with either pre-synthesized RNA molecules or plasmid DNAs encoding the chimeric inhibitor RNAs. 

## 2. Results and Discussion

### 2.1. In vitro HIV-1 RNA cleavage by anti-HIV-1 catalytic RNAs

Hairpin (HP) and hammerhead (HH) ribozymes were designed to target sequences 107-120 and 153-166 of the NL4.3 HIV-1 strain, which we had previously shown as being sequences accessible to both kinds of ribozyme [18]. Engineered HH ribozymes cleave at the 3’ side of nucleotides 114 and 160, while HP ribozymes cleave at the 3’ side of nucleotides 111 and 157 (Figure 1C). The sequence of these ribozymes and their targets is represented in Figure 1 and we will refer to them as HH113 and HP113 for those targeting the 107-120 region (they cleave surrounding position 113) and HH159 and HP159 for those targeting the 153-166 region (they cleave surrounding position 159).

The DIS region is a 34 nt-long RNA domain folded into a stem-loop structure that expands from nucleotide position 244 to 278 in the NL4.3 HIV-1 strain. A molecule containing the 23 most apical nucleotides of the DIS stem-loop, positions 249 to 271, was used as the decoy domain (DIS). A molecule having the complementary sequence of the same 23 nt was used as the antisense domain (αDIS; Figure 1B). The decoy or the antisense molecules were covalently linked to the 3’ end of HH113, HH159, HP113 and HP159 ribozymes to yield a series of potential HIV inhibitors named HH or HP-DIS or αDIS when carrying the sense or the antisense sequence, respectively (Figure 1C). In addition, the stem-loop flanking domains of the U6 snRNA were added to either end of the inhibitor RNA molecule to make them of the same structure as those to be assayed in the culture cells. For anti-viral activity assays, RNA chimeras were cloned into a mammalian pcDNA3-derived plasmid containing a U6 snRNA promoter cassette which directs the synthesis of inhibitor RNAs [19]. Transcription from this promoter yields RNA products flanked by the first and the last stem-loops of the U6 snRNA (Figure 1E).

**Figure 2:**
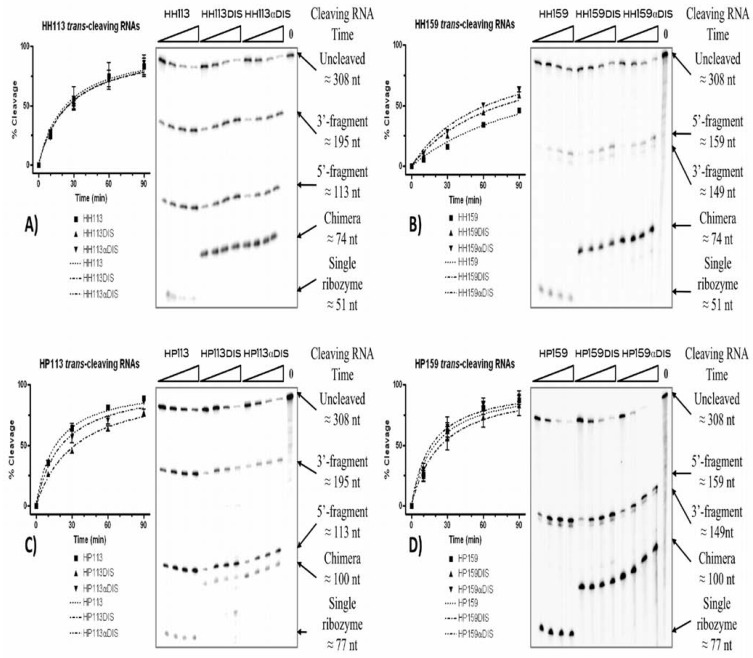
Cleavage reactions of 5’UTR by ribozymes and chimeric RNA molecules. Each gel autoradiograph shows a representative cleavage reaction time course of a specific ribozyme and its respective DIS and αDIS derived chimeras: **(a)** HH113, **(b) **HH159, **(c)** HP113 and **(d) **HP159. Each experiment was performed in triplicate and the cleavage percentage was obtained by quantification of the cleavage products. Curves were fitted with maximum sqr(R) coefficients.

*In vitro* cleavage activity of each chimera RNA was assayed and compared to that of the corresponding trans-cleaving ribozyme lacking any additional domain (Figure 1D and E). An *in vitro *transcribed HIV-1 5’UTR molecule containing the first 308 nt was used as the substrate of the reaction. Cleavage reactions were performed at 37 ºC in a cleavage buffer and the reaction progression was monitored at different times (Table 1 and Figure 2). All RNAs follow a hyperbolic pattern of cleavage percentage during the reaction time, with a 100% cleavage expected from all of them with a lowest r^2^ coefficient of 0.8824. We defined the T_0.5_ coefficient as the time, in minutes, that was necessary to reach 50% of the cleaved substrate. The hairpin ribozymes HP113 and HP159 had lower T_0.5 _values, 16.25 ± 2.17 and 19.47 ± 6.05 min, respectively, compared to their hammerhead counterparts, HH113 and HH159 with 22.89 ± 9.89 and 118.9 ± 39.54 min, respectively (Table 1).

The addition of either sense or antisense DIS domains resulted in a slight modification of the T_0.5_ value with respect to the one of the ribozyme alone (Table 1). The highest improvement was recorded for HH159 with the addition of the αDIS domain, which resulted in a nearly two-fold reduction of the T_0.5_. On the opposite side, the addition of the DIS domain to the HP113 ribozyme produced a two-fold increase of the T_0.5_.

**Table 1:** *In vitro* cleavage reactions of the HIV-5’ UTR by catalytic RNAs. ^(a)^T_0.5 _values were normalized to the one of the corresponding ribozyme. ^(b) ^Regression curves were fixed to a maximum cleavage value of 100%.

RNA	Maximum cleavage (%)	T_0.5_ (min)	Relative T_0.5_^(a)^	sqr(R)^ (b)^
HH113	100 ± 14.23	22.89 ± 9.88	1	0.8824
HH113DIS	100 ± 10.21	24.92 ± 7.43	1.08	0.9417
HH113αDIS	100 ± 7.99	25.4 ± 5.88	1.11	0.9667
HP113	100 ± 3.78	16.25 ± 2.17	1	0.9863
HP113DIS	100 ± 6.15	32.34 ± 5.17	1.98	0.9823
HP113αDIS	100 ± 5.67	20.99 ± 3.75	1.28	0.9746
HH159	100 ± 21.26	118.9 ± 39.54	1	0.9764
HH159DIS	100 ± 10.94	75.48 ± 15.15	0.63	0.986
HH159αDIS	100 ± 11.84	62.25 ± 14.57	0.52	0.9783
HP159	100 ± 9.53	19.47 ± 6.05	1	0.9369
HP159DIS	100 ± 14	24.44 ± 10.08	1.25	0.8942
HP159αDIS	100 ± 9.1	16.61 ± 5.28	0.85	0.9322

We previously reported that the addition of a TAR antisense domain (αTAR) to the same HP and HH ribozymes used in this work, resulted in an improvement of the cleavage activity [18]. The TAR RNA shares common features with natural antisense RNAs and this could explain why the αTAR-TAR interaction was able to mimic the fast and stable interactions of natural antisense RNAs and their targets, thus explaining the reported improvement in catalytic activity [18,20,21]. Neither DIS nor αDIS domains conserved the features of natural antisense, which may explain the lack of cleavage activity enhancement.

### 2.2. In vitro inhibition of the 5’UTR dimerization by catalytic RNAs

The HIV-1 virion particles contain two genome RNA copies. The process by which two genome copies get non-covalently bond is called dimerization and it is initiated at the DIS domain located at the 5’-end of the genomic RNA [22]. The dimerization process can be reproduced *in vitro* in a high salt concentration and can be resolved by native gel electrophoresis [23].

**Figure 3:**
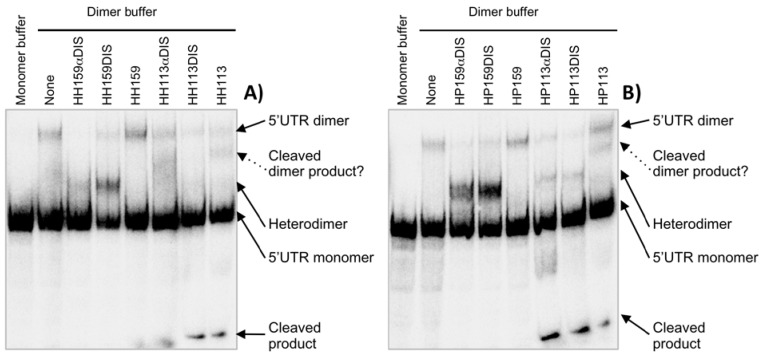
Inhibition of 5’UTR dimerization *in vitro* by chimeric inhibitor RNAs derived from both hammerhead and hairpin ribozymes [**A**) and **B**), respectively). The 5’UTR dimerization was performed in the presence and absence of inhibitor RNAs. Dimerization reactions were carried out at 37 ºC for 20 min with 157 nmol of internally radiolabelled 5’UTR RNA and a 20-fold excess of inhibitory RNA. The reactions were resolved in 4% polyacrylamide native gels. Line 1 in both gels represents non-dimer condition controls. Line 2 represents 5’ UTR dimerization in the absence of any RNA inhibitor. The shifted band within line 2 with respect to line 1 is considered as the dimer.

To check whether the inhibitory chimera RNAs might interfere with the dimerization process, both molecules (inhibitory RNA and 5’UTR) were mixed together in H_2_O, using a 20-fold molar excess of non-radiolabelled inhibitory RNA. The dimerization reaction was started by the addition of a reaction buffer. The dimer fraction was detected as the molecular species showing a slower mobility in a native polyacrylamide gel electrophoresis. The RNA chimeras based on HH159 and HP159 clearly interfered with dimer formation whereas ribozymes HH159 and HP159 did not have any effect. These chimeras led to an almost complete suppression of the dimer in favour of the appearance of a new species which migrates between the dimer and the monomer. This new species might be a heterodimer formed between the inhibitory RNA and the 5’UTR, although we cannot rule out other possibilities (Figure 3).

Similarly, the HH113 and HP113 chimeras also suppressed dimer formation almost completely and yielded a new species, supposedly a heterodimer, which was detected between the dimer and the monomer. Surprisingly, ribozymes HP113 and HH113 induced the appearance of a new molecular species that migrates between the heterodimer and the 5’UTR-5’UTR homodimer and a second species that migrated faster in the gel. Interestingly, catalytic RNAs targeting position 113 were the only inhibitors able to cleave the substrate under dimerization conditions (data not shown). It is feasible that the new species detected might correspond to a partially or completely cleaved dimer, which would explain the disappearance of the dimer species in the presence of HH113 (Figure 3). Likewise, 113 ribozymes interacting with 5’UTR cleavage region might switch the equilibrium towards the LDI conformer. It was previously reported that mutations in unrelated regions like TAR switch the 5’UTR isoform equilibrium towards one or the other of the conformers [24]. It is worth noting that the HH ribozymes used in this work maintained a perfect base pairing with the target sites within the 5’ UTR sequences. These target sequences completely met the HH defined consensus requirements. However, one mismatch existed between the binding arms and the target site for HP113, and two for HP159, which could affect HP159 cleavage efficiency under dimerizing conditions (Figure 1C). Furthermore, position 159 was predicted to be involved in a stem-loop structure in both BMH and LDI conformers while position 113 only appeared as single stranded in the BMH form, which could explain why 113 ribozymes were able to cleave the target despite non-optimal conditions while 159 ribozymes were not. 

### 2.3. Inhibition of HIV-1 ex vivo viral production by endogenously synthesized inhibitory RNAs

A HEK293T cell system was used for assaying the effect of inhibitor RNAs on HIV viral production. The HEK293T cells do not express the surface receptors required for HIV-1 infection; the virus can replicate in these cells but viral particles cannot infect them. Transfection with pro-viral DNA mimics the post-integration stage of the viral cycle and viral particles in the supernatant after transfection correspond to first generation virions.

**Figure 4:**
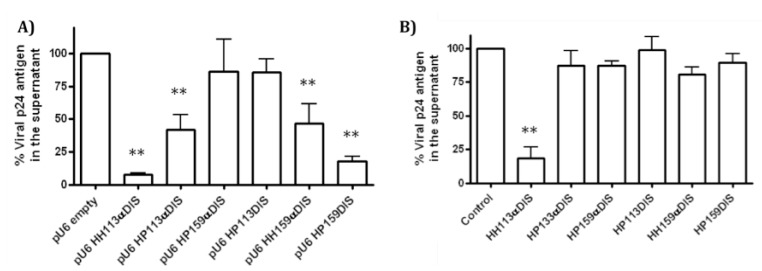
*Ex vivo* inhibition of HIV-1 viral production. The HEK293T cells were transfected with the pNL4.3 plasmid and an inhibitory RNA expressing vector at a molar ratio of 1:10 **A**) or with an *in vitro* synthesized RNA **B**). The bar graph represents the percentage of viral protein p24 levels in the supernatant 48 hours after transfection relative to the control. The inhibitory RNA is indicated below each bar. The empty pU6 vector and an RNA molecule consisting of both U6 snRNA flanking hairpins without any sequence in between were used as negative controls in **A**) and **B**), respectively. The results are the means of three independent experiments (** p < 0.01).

Inhibitor RNAs were cloned in a pcDNA3-derived plasmid, pU6, in which both CMV promoter and BGH polyadenylation signals were substituted by those belonging to human U6 snRNA [16]. The pU6 plasmid constructs encoding HH113DIS and HH159DIS RNAs were not obtained. The HEK293T cells were co-transfected with pNL4.3 and a pU6-inhibitor RNA. Intracellular expression of the pro-viral DNA encoded in the pNL4-3 plasmid allowed the viral replication cycle. The pU6-construct series allowed endogenous production of the inhibitor RNAs to be tested. Viral production was determined as a measurement of the viral protein p24 in cellular supernatant two days after transfection. The HH113αDIS and HP159DIS RNAs reached extracellular protein p24 inhibition rates of 92.68 ± 1.31% and 83.64 ± 3.78%, respectively. The HP113αDIS and HH159αDIS RNAs reached inhibitions rates close to 50%, while HP159αDIS and HP113DIS had no significant effect (Figure 4A). It was previously reported that the expression of single ribozymes does not interfere with HIV-1 viral production under these conditions [16]. 

### 2.4. Inhibition of HIV-1 viral production ex vivo by pre-synthesized inhibitory RNAs

To further characterize the inhibitory efficiency of these RNAs, a similar experiment to the one described above was performed, but this time *in vitro* synthesized inhibitory RNAs were transfected. These RNAs were transcribed *in vitro* by T7 RNA polymerase from PCR templates obtained by amplification from the pU6 constructs. The templates were designed to obtain RNAs containing both U6 snRNA flanking hairpins in order to be identical to those produced by the U6-driven expression cassettes (Figure 1F). As a control, an RNA consisting of the U6 snRNA flanking hairpins without any sequence in between was used.

The HEK293T cells were co-transfected with pNL4.3 and inhibitory RNAs. Viral protein p24 levels in supernatant were measured 48 hours after transfection. Up to 80% inhibition was obtained with HH113αDIS RNA, a similar value to the one obtained when it was endogenously synthesized from the U6 construct derivative. However, no significant effect was observed for the other tested RNAs (Figure 4B).

### 2.5. HIV-1 inhibition by catalytic RNAs is not explained by the interferon response

**Figure 5:**
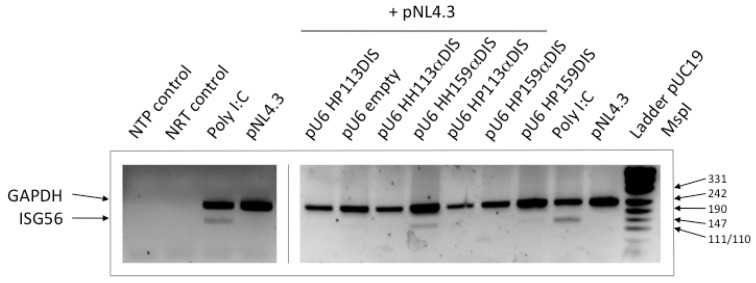
Assay of the interferon response. A representative photograph of an agarose gel visualized by ethidium bromide transluminescence is shown. Transfected DNAs are indicated on top of each lane. The NTP, control PCR reaction in the absence of any template. NRT, PCR reaction from total RNA without previous reverse transcription were checked for genomic DNA contamination. The poly I:C was a positive control of the interferon response. The amplicons are identified by the name of the gene on the left. Sizes of marker DNA fragments in bp are indicated on the right.

Total RNA was extracted from HEK293T cells co-transfected with pU6 constructs and pNL4.3 at the moment of quantifying viral protein p24 in the supernatant. The RNA was reverse transcribed using random hexamers and cDNA as a template for PCR, using specific primer pairs for the GAPDH and ISG56 genes. The interferon stimulated gene 56 (ISG56) is a gene induced by double-stranded RNA and virus infection [25,26]. Basal expression levels are nearly undetectable. Semiquantitative RT-PCR was performed to detect ISG56 mRNA as reporter of interferon-signalling activation using the housekeeping GAPDH gene as the internal control (Figure 5). Poly I:C is a double-stranded RNA that quickly triggers the interferon response; it is used as a positive control of interferon response induction. Semi-quantitative RT-PCR from total RNA extracted from poly I:C transfected cells generated a detectable ISG56 amplification. On the contrary, RT-PCR from total RNA extracted from cells solely transfected with pNL4.3 did not yield any ISG56 amplification products (Figure 5). 

Amplification of the ISG56 gene was not observed in any RT-PCR from total RNA extracted from cells co-transfected with pNL4.3 and any of the following pU6 construct derivatives: empty vector, HP113DIS, HH113αDIS, HP113αDIS or HP159αDIS (Figure 5). Only interferon signalling was slightly induced by the pU6 HH159αDIS construct and to a lower extent by pU6 HP159DIS (Figure 5). These results indicate that the highest viral inhibition recorded by pU6 HH113αDIS and pU6 HP159DIS cannot be explained by the induction of interferon-signalling, suggesting that the observed inhibition corresponded mainly to specific inhibition resulting from the direct action of the inhibitor RNAs on viral RNA. Nor was the viral inhibition produced by HP113αDIS due to the induction of interferon signalling.

### 2.6. Relationship between the HIV-1 RNA dimerization process and the structural conformation of the 5’UTR

**Figure 6:**
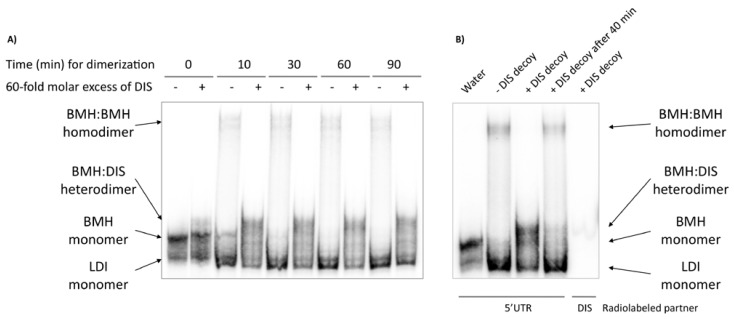
Involvement of the different 5’UTR conformers in the RNA dimerization process. (**A**) Autoradiogram of a polyacrylamide gel electrophoresis of a 5’ UTR dimerization reaction time course. Dimerization reactions were performed as described in the experimental section. Different molecular species are identified at the left side of the gel. (**B**) The thermodynamic relationship between 5’UTR RNA isoforms. Line 1 (water): 5’UTR was denatured and renatured in water. Line 2 (–DIS decoy): 5’UTR was denatured and renatured in water, and incubated for 40 min at 37 ºC in 1X dimer buffer. Line 3 (+DIS decoy): the same procedure as line 2, but a 60-fold molar excess of unlabelled DIS decoy was added at time 0. Line 4 (+DIS decoy after 40 min): the same procedure as line 2 but a 60-fold molar excess of unlabelled DIS decoy was added after 40 min incubation in dimerization conditions; the reactions were followed for another additional 40 min at 37 ºC. Line 5 (+DIS decoy): the same procedure as line 2, but the DIS decoy was internally radiolabelled and 5’UTR unlabelled. Different molecular species are identified by a schematic representation depicted on the right side of the gel.

The monomeric conformers LDI and BMH could not be resolved under the dimerization conditions tested (Figure 6A, lines 1 and 2 from the left of each gel). However, the internally labelled 5’ UTR in the absence of any reaction buffer was resolved in a native polyacrylamide gel using 0.25X TBE in the gel and the running buffer, and a new conformer showing a slightly retarded mobility was observed. (Figure 6A, first line). We associated the fast and slow migrating monomers to LDI and BMH forms, respectively. Ionic strength stabilized the highly imperfect LDI helices, while conserved BMH hairpins were favoured in their absence. The monomer detected under dimerization conditions might correspond to the LDI conformer (fast migration). A dimerization reaction time course was performed to study the progression of the reaction (Figure 6A, odd lines). The BMH form extinguished between 10 and 30 min after dimerization started, while maximum dimerization was reached at no later than 30 min (Figure 6A). Only a minor percentage of the 5’UTR RNA population formed a dimer. This suggests that once the salt is added to the mix, the 5’UTR BMH conformer quickly switches to the LDI conformer, and only a minor proportion of molecules progress to dimer formation. This might explain why these inhibitor chimera RNAs with DIS or αDIS did not behave like αTAR ones, increasing the percentage of cleaved molecules *in vitro*. The TAR is present within both LDI and BMH conformers, while the DIS structure is only present in a very limited percentage of the 5’UTR molecules. 

To further characterize the anti-HIV dimerization effect of the inhibitory RNAs, a DIS decoy RNA was used as a competitor. The DIS decoy might stabilize BMH within a heterodimer and could be a tool for the identification of different isoforms and for understanding their equilibrium relationships. Dimerization reactions were performed in the presence of a 60-fold molar excess of a cold 23 nt-long DIS decoy, which was added to the mix just before the reaction began. The DIS decoy completely abolished dimer formation, while a new RNA complex appeared which migrated slightly slower than the BMH monomers (Figure 6A, even lines). The same experiment was performed using cold 5’UTR and a radiolabelled DIS RNA decoy, and revealed that the decoy is only present in the new slow migrating complex, so this new molecular species must be an 5’UTR-DIS heterodimer (Figure 6B, fourth line). These results suggest that the DIS decoy sequesters the dimerization-competent BMH conformer from natural dimerization. However, no competition was observed when the DIS decoy was added 40 min after the reaction started (Figure 6B). This indicates that the BMH homodimer is stable enough and a dynamic equilibrium does not exist between the dimer and the LDI monomer via the ephemeral BMH. This phenomenon explains why the 5’UTR was able to dimerize by the BMH-dependent DIS domain, despite the large thermodynamic displacement toward the LDI form. In any case, the chaperone activity of the nucleocapsid protein could probably displace the equilibrium towards the dimer formation and explain the BMH conformer predominance obtained by *ex vivo* probing assays [3].

## 3. Experimental

### 3.1. RNA preparation and vector construction

Inhibitor RNAs were obtained by *in vitro* transcription from appropriate DNA templates using bacteriophage T7 RNA polymerase. The DNA templates were generated from pG3HH and pG3HP anti-LTR HIV-1 plasmid series as described by Puerta-Fernández and co-workers [18] by replacing the antisense TAR coding sequence by either a DIS or an antisense DIS (αDIS) coding sequence. Briefly, DIS and αDIS coding sequences were obtained by annealing DISf (5’GATCTCTTGCTGAAGCGCGCACGGCAAGG3’) and DISr (5’GATCCCTTGCCGTGCGCGCT TCAGCAAGA3’) oligonucleotides or αDISf (5’GATCTCTTGCCGTGCGCGCTTCAGCAAGG3’) and αDISr (5’GATCCCTTGCTGAAGCGCGCACGGCAAGA3’) oligonucleotides, respectively. The oligonucleotides were 5’ end phosphorylated by T4 polynucleotide kinase (Roche, Switzerland) before annealing, following the manufacturer’s instructions. Each pG3HH and pG3HP construct was digested with *Bgl*II and *Bam*HI restriction enzymes to eliminate the antisense TAR domain coding sequence and ligated with the DIS inserts, yielding the new pG3HH and pG3HP series encoding anti-DIS sequences. *In vitro* transcription was performed as previously described [18] from *Bgl*II or *Bam*HI linearized pG3HH and pG3HP plasmid templates to synthesize ribozymes or inhibitor chimera RNAs, respectively.

*Ex vivo* expressing plasmid constructs were generated from the pU6-Rzs plasmid series as described previously [16]. Inhibitor RNA coding sequences were obtained by PCR amplification using the constructs described above as templates, and the oligonucleotides 5’KpnIpK (5’GACTCGGTACCGGGCGAATT3’) and 3’ApaIpK (5’TCTAGAGGGCCCCCTTGC3’). Amplicons were digested with *Kpn*I and *Apa*I and cloned in the *Kpn*I and *Apa*I unique restriction sites within the U6 promoter cassette of the pU6-Rzs plasmid series. 

Pre-synthesized RNAs for *ex vivo* HIV-1 inhibition assays were generated by *in vitro* transcription with T7 RNA polymerase from PCR amplicons. Templates were generated from the pU6 constructs using 5’T7U6 (5’TAATACGACTCACTATAGGGGTGCTCGCTTCGGCAGCACAT3’) and 3’ApaIU6 (5’AGCGGGCCCAAAAAGCGGACCGAAGTCCGC3’) oligonucleotides which contained the T7 RNA polymerase promoter at the 5’ end. Transcription from these templates yielded chimera RNAs containing U6 snRNA flanking hairpins at both ends, as they would be generated by *ex vivo* transcription. 

The 5’UTR RNA was transcribed *in vitro* by T7 RNA polymerase from a PCR template obtained from the pNL43 plasmid (GeneBank no. AF324493) with primers 5’T7NL43 (5’TAATACGACTCA CTATAGGGTCTCTCTGGTTAC3’) and 3’T7NL43 (5’AATTTTTGGCGTA CTCACCAGT3’). The amplicon incorporated the T7 RNA polymerase promoter at the 5’-end of HIV-1 genomic region from the +1 to +308 nucleotide coding sequence.

### 3.2. In vitro cleavage assays

The cleavage reactions were performed in a 10 µL final volume in the presence of 0.2 nmol of internally radiolabelled 5’UTR RNA and 20 nmol of non-radiolabelled catalytic inhibitor RNAs. The RNA mix was denatured (10 min 65 ºC) and renatured (10 min 37 ºC) in H_2_O and the reaction was initiated by the addition of 2 µL of 5X cleavage buffer (1X: Tris-HCl 50 mM pH 7.5; MgCl_2_ 10 mM) and incubated at 37 ºC. Aliquots were taken at different times (10, 30, 60 and 90 min) and the reaction stopped by adding an equal volume of 2X denaturing loading buffer (2X: deionized formamide 94% v/v; xylene cyanol 0.025% p/v; bromophenol blue 0.025% p/v; EDTA 17 mM). The results were resolved in 8% denaturing polyacrylamide gels in 1X TBE using 0.5X TBE as the running buffer. 

### 3.3. In vitro dimerization assays

Dimerization competition reactions were performed in a final volume of 10 µL. Internally radiolabelled 5’UTR RNA (157 nmol) was mixed with non-radiolabelled inhibitory RNA (3.14 pmol) in 8 µL H_2_O. The RNA mix was denatured (10 min 65 ºC) and renatured (10 min 37 ºC) and the reactions were initiated by the addition of 2 µL of 5X dimerization buffer (1X: sodium cacodylate 50 mM pH 7.5; KCl 0.3 M; MgCl_2_ 5 mM). The reactions were performed for 20 min at 37 ºC and then stopped by the addition of a same volume of 2X native gel loading buffer (2X: Tris-acetate 20 mM; Mg acetate 10 mM; NaCl 0.1M; glycerol 30% v/v; xylene cyanol 0.4% p/v; bromophenol blue 0.4% p/v; tRNA 4% p/v). The reaction products were resolved by 4% native polyacrylamide gel electrophoresis in 1X TBE using 1X TBE as the running buffer. Electrophoresis was performed at 4 ºC and no more than 15 mA per gel. Monomerization control was performed by the addition of 5X monomerization buffer (1X: sodium cacodylate 50 mM pH7.5; KCl 0.3 M; MgCl_2_ 0.1 mM). For detection of the 5’UTR conformers BMH and LDI, the dimerization mix contained 0.1 µmol of 5’UTR and 6 µmol of DIS decoy, and the reactions were resolved in 4% native polyacrylamide gels in 0.25X TBE using 0.25X TBE as the running buffer.

### 3.4. HIV-1 ex vivo inhibition assays

For these assays 250,000 HEK293T cells per well were plated in a 24-well plate using 500 µL/well of DMEM, 10% fetal bovine serum (FBS) and L-glutamine 2 mM the day before transfection. Then, 1 µL of Lipofectamine 2000 reagent (Invitrogen, CA, USA) was mixed with 25 µL of Opti-MEM^®^ (PAA Laboratories GmbH, Austria) and incubated for 5 min at room temperature. Following this, 100 ng of pNL4.3 were mixed with either 300 ng of a pU6 DNA plasmid construct coding for a specific RNA inhibitor or 500 ng of a pre-synthesized inhibitory RNA in 25 µL of Opti-MEM^®^, and incubated for 5 min. Lipofectamine and nucleic acid solutions were mixed and incubated at room temperature for 20 min. Then, 450 µL of DMEM, 10% FBS and 2 mM L-glutamine were added. The supernatant was carefully removed from each well by aspiration and 500 µL transfecting-mix was added. Two days after transfection, the supernatants were removed and processed using the Genscreen HIV-1 Ag assay (BioRad, CA, USA) for viral protein p24 antigen quantification, following the manufacturer’s instructions. The DNA for transfection was prepared using the Plasmid Mini Kit (Quiagen, The Netherlands), following manufacturer’s instructions. Each treatment was assayed in triplicate and statistical analysis was performed by Kruskal-Wallis non-parametric test followed by multiple Dunnet comparison tests to check differences against the control.

### 3.5. Detection of interferon-response activation by semi-quantitative RT-PCR

Total RNA was extracted from the cells by the Trizol^®^ reagent (Invitrogen), following manufacturer’s recommendations but adding an extra incubation of the aqueous phase after chloroform extraction with 1 U of RQ1DNase (Promega, WI, USA) at 37 ºC for 15 min to prevent genomic DNA contamination. This was followed by an additional chloroform extraction. Reverse transcription was performed using the Reverse-Transcriptor kit (Roche), where 100 ng of total RNA was incubated at 37 ºC for 20 min with 0.5 U of RQ1 DNase (Promega) and 20 U of the RNase inhibitor (Promega). The RNA was incubated at 65 ºC for 10 min, then the temperature was brought down quickly to 4 ºC and 3.2 µg of random hexamer primers were added. The reaction mixes contained 1 mM dNTPs and 10 U of reverse transcriptase (RT) in reaction buffer in a final volume of 20 µL. Annealing was performed at 25 ºC for 10 min and the reactions were run at 60 ºC for one hour. Finally, the RT was inactivated at 85 ºC for 5 min. Semi-quantitative PCR was performed by the Reddy Mix PCR Master Mix (Thermo Scientific). The reactions were performed in a total volume of 10 µL containing 2.5 µL of cDNA, 3 pmol of each GAPDH-specific primer and 10 pmol of ISG56-specific primers. 

## 4. Conclusions

Nowadays, there are multiple RNA-based approaches with the therapeutic potential to interfere with viral functions. Functional RNA moieties have been involved in many regulatory processes. Understanding these functionalities, like RNA catalysis or HIV genomic RNA dimerization, may enable the design of therapeutic RNAs. Here we have proven that the combination of functional inhibitor RNA moieties directed against different HIV-1 5’UTR RNA targets in a single chimeric molecule results in bi-functional RNA molecules that fully conserve both inhibitory activities. Both hammerhead and hairpin ribozyme domains were explored as 5’UTR RNA trans-cleaving elements targeting two available cleavage sites surrounding the +113 and +159 positions. The DIS domain within the 5’UTR RNA, responsible for triggering the HIV-1 RNA dimerization, was used as target for a decoy and an antisense RNA.

The *ex vivo* HIV-1 inhibition assays demonstrated up to 90% effect measured as viral protein p24 levels in the supernatant of cells transfected with the HIV pro-viral DNA in a post-integrative model system. Inhibitory chimera RNAs achieved a complete *in vitro* inhibition of the essential viral dimerization process. A model of the implication of both 5’UTR folding isoforms LDI and BMH in the dimerization process is proposed. The results summarized here indicate that in the absence of ionic strength, the 5’UTR folds *in vitro* into the BMH monomeric conformer, which is competent for dimerization. This conformer quickly switches to the LDI form after the addition of ions. Under these conditions, a low proportion of folded BMH molecules can dimerize into a stable complex, ensuring the existence of enough HIV genome dimers for packaging and the generation of infective viral particles.
